# Manual Vacuum Aspiration Versus Expectant Management for First Trimester Miscarriage: A Randomized Controlled Trial

**DOI:** 10.7759/cureus.45731

**Published:** 2023-09-21

**Authors:** Sutharshan Ratnam, Sureshkumar Kulasingam, Abilashini Kanagallingam

**Affiliations:** 1 Obstetrics and Gynaecology, Postgraduate Institute of Medicine, University of Colombo, Colombo, LKA; 2 Obstetrics and Gynaecology, Teaching Hospital Jaffna, Jaffna, LKA

**Keywords:** complete evacuation in manual vacuum aspiration, miscarriage, complications of manual vacuum aspiration, manual vacuum aspiration success rate, expectant management, manual vacuum aspiration versus expectant management, randomized trial on manual vacuum aspiration

## Abstract

Background

Miscarriage is one of the common problems encountered in pregnancy. The treatment modalities are expectant, medical, and surgical management. This study compared the effectiveness and safety of manual vacuum aspiration (MVA) with expectant management for first trimester miscarriage.

Method

This randomized controlled trial was conducted in Teaching Hospital Jaffna, Jaffna, Sri Lanka, and 134 eligible patients with first trimester spontaneous miscarriage were randomized to expectant management (67) and MVA (67). Those allocated to expectant management were managed expectantly for up to two weeks, and those allocated to MVA underwent aspiration under a paracervical block in the ward. The primary outcome was complete evacuation of the uterus, and the secondary outcomes were duration of bleeding, duration of pain, level of pain, need for the second procedure, cervical or uterine injuries, and patient satisfaction.

Results

Of the 134 eligible women, seven were lost to follow-up and 127 were analyzed. The MVA was superior in achieving complete evacuation compared to expectant management (95.2% vs. 70.3%; p ≤ 0.001). Notably, in both groups, complete evacuation was more readily achievable in incomplete miscarriage than in missed miscarriage. Duration of bleeding (mean days, 1.6 vs. 4.3; p ≤ 0.0001), duration of pain (mean days, 1.0 vs. 4.2; p ≤ 0.0001), and the need for additional surgical procedure in the form of dilatation and curettage (4.8% vs. 29.7%; p ≤ 0.001) were lower in MVA. Patient satisfaction was higher in the MVA group than in the expectant group (93.7% vs. 65.6%; p ≤ 0.001). No statistically significant differences were observed between the groups in terms of blood transfusion and infection. There wasn’t any incidence of cervical damage or uterine perforation.

Conclusion

MVA is an effective and safe treatment method for first trimester miscarriage with higher patient satisfaction.

## Introduction

Miscarriage is a common event that can occur in any pregnancy and is seen in approximately 30% of all pregnancies [1]. Early fetal demise or missed miscarriage is characterized by minimal vaginal bleeding, abdominal pain, and loss of pregnancy symptoms with a closed cervix. Clinical features of incomplete miscarriage are partial passage of fetal parts with or without vaginal bleeding, pain, and open cervix. In contrast, complete miscarriage is characterized by cessation of vaginal bleeding and pain with a closed cervix [2]. An ultrasound scan, preferably a transvaginal scan, confirms the clinical diagnosis [3].

For the treatment of miscarriage, different modalities, such as expectant, medical, and surgical management, have been practiced worldwide. Expectant management denotes allowing some time for the natural expulsion of products without intervention. During this time, the patient may require rest, USS, and prophylactic antibacterial drugs to prevent infections. In studies done in the past, the success rate of expectant management varied from 58% to 80%, and complete evacuation of the product of conception was achieved within two weeks of recognition with minimal infection incidence [4-6]. Though expectant management has several advantages, such as avoidance of both side effects of medical management and complications of surgical management, it does have drawbacks, such as a more significant number of days of bleeding, the need for blood transfusion, unplanned hospital admissions, and sometimes demanding further interventions [7,8]. Surgical uterine evacuation is considered the gold standard management in spontaneous miscarriage because it is definitive, predictive, and relatively safe, with a success rate of 90-100% [9]. Surgical treatment for miscarriage can be either sharp curetting or vacuum aspiration; the latter can be either an electric or a manual vacuum aspiration (MVA). Conventionally, dilatation and curettage (DNC) is the choice of surgical management for miscarriage [10]. Albeit it is the quickest mode of remedy, it can result in uterine damage such as perforation, which necessitates secondary surgery, and intrauterine adhesions lead to the generation of scar or injury to the cervix that can result in cervical insufficiency during pregnancy [11]. On the other hand, MVA has emerged as better surgical management because of its effectiveness, safety with minimal complications, low cost, and additional benefits such as not requiring an operating theater with a skilled crew or general anesthesia [12-14]. MVA under local anesthesia as an office procedure is an emerging treatment modality for miscarriage in developing countries, and its success rate is equal to traditional surgical methods [15].

In Sri Lanka, though DNC and electric vacuum suction have been widely practiced as a surgical treatment for miscarriage, MVA has not been routinely practiced. In Sri Lanka, there haven’t been any studies comparing the effectiveness of MVA against other methods as a treatment of choice for early pregnancy demise. In most of the studies that assessed the treatment methods of miscarriage, the surgical technique was found to be the traditional DNC [16,17]. We conducted this trial to compare MVA to an office procedure with expectant management for first trimester miscarriage.

## Materials and methods

**Figure 1:**
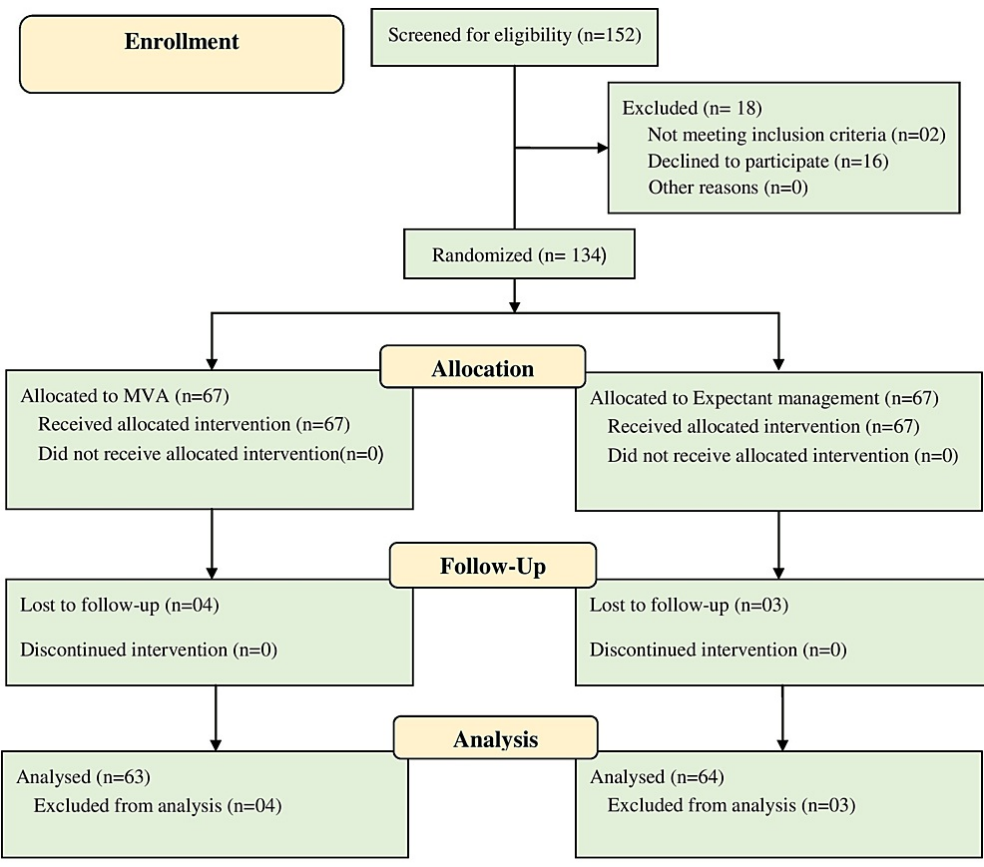
Flow of the participants through the stages of research MVA, manual vacuum aspiration

This randomized control trial was done in the obstetrics and gynecology ward in Teaching Hospital Jaffna, Jaffna, Sri Lanka, from January 2017 to January 2018. Ethical clearance was granted by the ethical review committee of the Faculty of Medicine, University of Jaffna, Jaffna, Sri Lanka. This trial was prospectively registered in the Sri Lanka Clinical Trials Registry (SLCTR/2017/014). The inclusion criteria of the study were as follows: pregnant women with the diagnosis of miscarriage presented with ≤12 weeks of gestation, hemodynamic stability, temperature not greater than 37.5°C, age 18 years or older, no history of using anticoagulants or steroids, and absence of significant medical or surgical conditions. Women diagnosed with pelvic infections, ectopic pregnancy, and bleeding disorders were excluded from the study. From the sample size calculation using 80% power, this trial required a sample of 62 in each arm, according to the anticipated loss (10%) of follow-up. A total of 152 participants were screened for eligibility, and 18 were excluded due to declining participation (16) and a history of pelvic infection (two). In all, 67 were allocated to each arm from the 134 eligible patients, and 127 were analyzed, as seven were lost to follow-up (Figure 1).

After the diagnosis and the fulfillment of the inclusion criteria, the participants were allocated to each arm of the trial according to the computer-generated random numbers. The sequentially numbered closed opaque envelope method was used to achieve allocation concealment, and investigators were not aware of the randomization schedule. Participants of each arm were explained about the treatment procedures and provided with symptom check sheets and information leaflets. Written informed consent was obtained before allocating to randomization. Clinical history and physical examination findings were documented. Complete blood count, blood grouping, and rhesus typing were done. They were instructed to return to the hospital if they developed any heavy vaginal bleeding, fever, or severe abdominal pain and were provided with the contact number of the instructor. Heavy vaginal bleeding was defined as soaking two pads in one to two hours, persistent body temperature equal to or more than 99.5°F (or 37.5°C) for four hours was considered a fever, and severe abdominal pain was considered a scale of 8-10 in the visual analog score (VAS). During the follow-up visits, all women were assessed clinically in view of complete evacuation and complications and underwent transvaginal ultrasound regardless of clinical findings. The same experienced medical officer performed all the transvaginal scans.

Women in the expectant management arm were allowed home after the initial assessment with symptoms check sheets, and they were instructed to return to the hospital if they developed any heavy vaginal bleeding, fever, or severe abdominal pain before the scheduled follow-up visits at days 7 and 14 in gynecology outpatient clinic or ward. They were advised to take paracetamol for the pain, on a demand basis.

Women allocated an MVA arm were given a stat dose of oral antibiotics (azithromycin 1 g), paracetamol (1 g), and ibuprofen (400 mg) one hour before the procedure. Rhesus negative women were administered anti-D prophylaxis. Registrars and senior medical officers who have prior experience in MVA performed the procedure, which was done under the paracervical block with 2% lignocaine using a handheld MVA pump. Patients were observed for six hours post-procedure and discharged home if hemodynamically stable. They were instructed to return to the hospital if they developed any heavy vaginal bleeding, fever, or severe abdominal pain before the scheduled follow-up visit one-week post-procedure in the gynecology outpatient clinic or ward. The primary outcome of the research was the rate of complete emptying of the uterus. The completeness was defined as an empty uterus with an endometrial thickness below 15 mm calculated in the anterior-posterior direction on transvaginal ultrasound examination [3]. In each arm, those who failed to complete the uterus emptying were scheduled for surgical evacuation under general anesthesia in the theater as a day case.

Secondary outcome measures were bleeding duration, pain duration and level, unplanned surgical evacuation, blood transfusion, pelvic infection, uterine or cervical damage, and patient satisfaction. A combination of Interviewer-administered questionnaires and patients’ symptom check sheets were used to collect the demographic and clinical details. Patient satisfaction was assessed by questions included in the questionnaire. Participants were asked, “overall, how will you rate your experience with the treatment that you received?” responses on a two-point scale “I am satisfied” or “I am not satisfied.” The infection was diagnosed clinically and biochemically in suspected patients, and all adverse effects were managed as per ward protocol. Statistical data were analyzed using IBM SPSS Statistics, version 21.0 (IBM Corp., Armonk, NY). One-way ANOVA or student’s t-test was used to analyze the differences in the continuous variables. The differences in proportions were analyzed by the chi-square test. A p-value of ≤0.05 was acknowledged to be statistically significant.

## Results

Table 1 presents the demographic and obstetric information, and no statistical differences were found among the two treatment groups.

**Table 1:** Demographic and obstetric characteristics of the study population MVA, manual vacuum aspiration; BMI, body mass index; SD, standard deviation; NS, not significant

	MVA (N = 63)	Expectant management (N = 64)	p-value
Age (year), mean ± SD	29.8 ± 5.0	30.0 ± 5.3	NS
Range	19-40	18-42	
BMI (kg/m^2^)			
Mean ± SD	24.8 ± 3.6	24.0 ± 3.1	NS
Range	17.5-34.5	18.8-33.5	
Planning of pregnancy, n (%)			
Planned	62 (98.4%)	62 (96.9%)	NS
Unexpected	01 (1.6%)	02 (3.1%)	NS
Previous miscarriage, n (%)	08 (12.7%)	15 (23.4%)	NS
Gestational age (days)			
Mean ± SD	68.9 ± 9.9	71.2 ± 7.3	NS
Range	58-84	49-84	
Parity, n (%)			
Nullipara	25 (39.7%)	29 (45.3%)	NS
Multipara	38 (60.3%)	35 (54.7%)	NS
Ultrasound diagnosis, n (%)			
Missed miscarriage	48 (76.2%)	44 (68.8%)	NS
Incomplete miscarriage	15 (23.8%)	20 (31.2%)	NS

**Figure 2:**
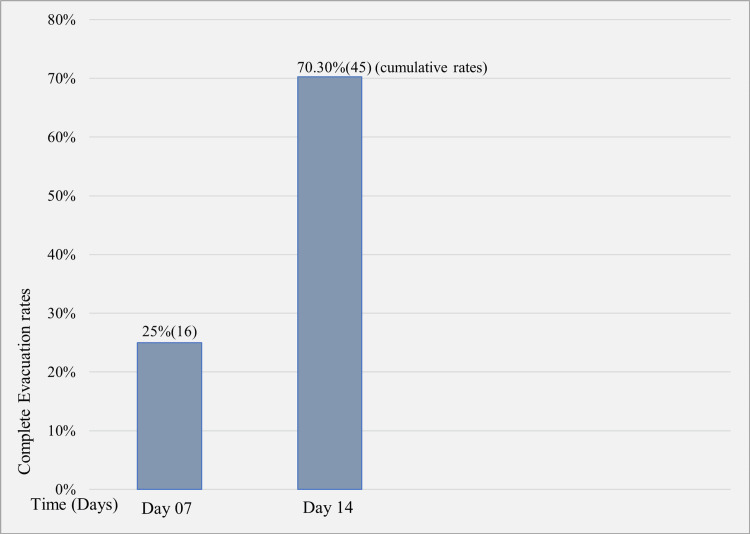
Success rate of expectant management according to time

The complete evacuation of the uterus was achieved in 95.2% (60/63) of patients in the MVA group and 70.3% (45/64) of patients in the expectant management group (Table 2). Among the 45 patients who had complete evacuation in the expectant management group, 16 (25%) achieved it by day 7, and the remaining 29 (45.3%) succeeded by day 14 (Figure 2). When considering the type of miscarriage, the success rate was 100% (15/15) in incomplete miscarriage and 93.75% (45/48) in missed miscarriage in the MVA group, whereas it was 90% (18/20) and 61.35% (27/44) in the expectant management group, respectively (Table 2). Three patients from MVA and 19 patients from the expectant management group failed to achieve complete evacuation and underwent surgical evacuation in the operation theater under general anesthesia in the allocated theater list for the unit.

**Table 2:** Primary outcomes of manual vacuum aspiration and expectant management MVA, manual vacuum aspiration; NA, not applicable

	MVA (N = 63)	Expectant management (N = 64)	p-value
Overall success rate	95.2% (60/63)	70.3% (45/64)	≤0.0001
Success rates according to the type of miscarriage at diagnosis			
Incomplete miscarriage	100% (15/15)	90% (18/20)	NA
Missed miscarriage	93.75% (45/48)	61.35% (27/44)	NA

Table 3 presents the secondary outcomes. The duration of bleeding and pain was less in the MVA group. The VAS (0-10) was used to rate the pain and categorized it into four groups: no pain (0), mild (1-3), moderate (4-7), and severe (8-10). In the MVA group, most of the patients, 69.8% (n = 44), experienced mild pain after the procedure.

**Table 3:** Secondary outcomes GA, general anesthesia; VAS, visual analogue score; MVA, manual vacuum aspiration; SD, standard deviation; NS, not significant

	MVA (N = 63)	Expectant management (N = 64)	p-value
Duration of bleeding (days)			
Mean ± SD	1.6 ± 1.5	4.3 ± 1.0	≤0.0001
Range	1.0-7.0	1.0-8.0	
Duration of pain (days)			
Mean ± SD	1.0 ± 0.8	4.2 ± 1.0	≤0.0001
Range	1.0-4.0	1.0-7.0	
Level of pain by VAS, n			
No pain (0)	0	0	
Mild (1-3)	44	26	≤0.001
Moderate (4-7)	15	33	≤0.001
Severe (8-10)	04	05	NS
Surgical evacuation under GA, n (%)	03 (4.8%)	19 (29.7%)	≤0.001
Blood transfusion, n (%)	01 (1.6%)	02 (3.1%)	NS
Infection, n (%)	01 (1.6%)	03 (4.7%)	NS
Uterine perforation, n (%)	0%	0%	-
Cervical trauma, n (%)	0%	0%	-
Patients’ satisfaction, n (%)	59/63 (93.7%)	45/64 (65.6%)	≤0.001

The need for the second procedure for the uterine evacuation with DNC was less in the MVA group when compared to expectant management (Three vs. 19). The rate of blood transfusion and infection events did not differ statistically between the groups, although they were found to be less in the MVA group. There was no incidence of uterine perforation or cervical damage. Patient satisfaction was higher in the MVA method (93.7% vs. 65.6%; p ≤ 0.001).

## Discussion

This trial has compared the effectiveness and safety of MVA with expectant management in patients with first trimester pregnancy loss. Based on our study, it has been shown that MVA had a statistically significant success rate in achieving complete evacuation when compared to expectant management (95.2% vs. 70.3%; p ≤ 0.0001). It has been found that evacuation with MVA is more effective in the incomplete miscarriage type than in missed miscarriage (100% vs. 93.75%). In the past, the effectiveness of MVA was compared with electric vacuum aspiration (EVA) and traditional DNC, and the effectiveness of MVA in our study was comparable to the results of the previous studies [13-15,18,19].

Our trial has achieved complete evacuation in 70.3% (45/64) of patients in the expectant management arm. In contrast, the other studies’ overall success rate of expectant management varied from 44.2% to 94.4%, and this wide variation could be due to factors such as the type of miscarriage and duration of follow-up [16,20,21]. As has also been shown in other studies, complete evacuation was readily achievable in incomplete miscarriage compared to missed miscarriage (90% vs. 61.35%) and with a longer waiting period (Figure 2).

Surgical procedures clear the retained products quicker than expectant management, where the uterus naturally contracts and evacuates its contents, possibly resulting in more prolonged bleeding. In our trial, the duration of vaginal bleeding experienced by the MVA group was smaller than the expectant management group (mean days, 1.6 vs. 4.3; p ≤ 0.0001). Similar results were found in the previous studies that compared surgery vs. expectant management; however, the surgical management of these studies was the traditional DNC [17,21].

In the present study, patients in the MVA group experienced pain for less duration compared with the expectant group (mean days, 1.0 vs. 4.2; p ≤ 0.001). A study comparing surgical vs. expectant management done by Wael et al. showed a comparable, statistically significant, and higher number of days of pain (mean days, 5.5 vs. 8.1) in both groups. However, the surgical management in that study was DNC [21]. Pain is a subjective parameter and challenging to quantify. Most of the patients (69.8%) in the MVA group experienced mild pain, and severe pain was experienced by 6.3% of the MVA patients. In the expectant management group, the percentage of patients who experienced mild and moderate pain was nearly similar, 40.6% and 51.5%, respectively. Among those who had severe pain, one from the MVA group and two from the expectant management group were found to have pelvic infections and received appropriate treatment. Our study has demonstrated comparable results in terms of level of pain for MVA, as has been shown in a prospective descriptive study done on 456 patients to assess the impact of MVA on the health care system, in which patients complained of no pain and tolerable pain accounted for 75% combinedly [22].

The incomplete evacuation rate was less with MVA. In our study, only three (4.8%) from the MVA group and 19 (29.6%) patients from the expectant management group had incomplete evacuation and undergone surgical evacuation under general anesthesia. A study done by Milingos et al. on MVA showed a comparable rate of incomplete evacuation and required surgical evacuation (3.26%) [23]. In our study, one (1.6%) patient from the MVA group with incomplete emptying also had anemia and a low hemoglobin of 8.1 g/dL during the follow-up. Therefore, she received a blood transfusion and subsequently underwent surgical evacuation. Two (3.1%) patients from the expectant management group who had severe vaginal bleeding received one unit of blood transfusion before surgical evacuation. However, there was no statistical difference noted between the treatment groups.

Only one (1.6%) patient from the MVA group and three (4.7%) patients from the expectant management group were treated for infection with antibiotics. No statistical difference was noted between the groups in this study related to pelvic infections. The rate of infections in the MVA group in the present study was in concordance with the results of other studies [24,25]. Although there was insufficient data to recommend routine antibiotic prophylaxis before MVA, we used antibiotic prophylaxis in our trial, which could result in a smaller number of infections observed; therefore, the significance of the occurrence of infection cannot be commented on.

Many studies that compared the MVA with other surgical managements have proven that MVA is a safe procedure for treating miscarriage and found that MVA was associated with no or minimal serious complications such as cervical damage or uterine perforation [13-15,22,24]. In our study, none of the patients encountered uterine perforation or cervical trauma during MVA.

In evaluating patient satisfaction related to treatment modality, overall patient satisfaction was high with MVA compared to expectant management (93.7% vs. 65.3%). Other studies comparing MVA with EVA or with medical management found no significant differences in terms of patient satisfaction [26,27]. A study that compared expectant management vs. surgical has found that more dissatisfaction was associated with expectant management [4]. In the present study and other studies, the success rate of the treatment modality has been reflected in the patient satisfaction rates [4,28].

A cost-effective analysis was not included in this study. Based on the Annual Health Bulletin report of the Ministry of Health Sri Lanka 2015, 60% of the total gynecology-related hospital admissions were due to first trimester miscarriage. Among these, 78% of the patients underwent surgical management [29]. In Sri Lanka, surgical evacuation is the primary mode of treatment, done in theater suits with various equipment and under general anesthesia, and this would incur significant health expenditure. Therefore, including a cost-effective analysis in future studies might be helpful in utilizing the health resources productively.

Limitations

An estimation of vaginal blood loss was not performed as there are difficulties accurately measuring the amount of blood loss practically due to subjective variations in the visual estimation.

A cost-effective analysis could not be done due to practical difficulties.

## Conclusions

Our study has shown that MVA is an effective and safe method without significant complications and has a higher patient satisfaction rate. It can be done as an office procedure under local anesthesia and is considered a viable alternative to traditional surgical options for managing first trimester miscarriage after considering various factors, including patients’ preferences.
